# Amyloid-β 25-35 Induces Neurotoxicity through the Up-Regulation of Astrocytic System X_c_^−^

**DOI:** 10.3390/antiox10111685

**Published:** 2021-10-26

**Authors:** Veronica D’Ezio, Marco Colasanti, Tiziana Persichini

**Affiliations:** Department of Sciences, University “ROMA TRE”, 00146 Rome, Italy; veronica.dezio@uniroma3.it (V.D.); marco.colasanti@uniroma3.it (M.C.)

**Keywords:** Aβ_25-35_, Alzheimer’s disease, amyloid-β, astrocytes, Nrf2, oxidative stress, System X_c_^−^

## Abstract

Amyloid-β (Aβ) deposition, a hallmark of Alzheimer’s disease, is known to induce free radical production and oxidative stress, leading to neuronal damage. During oxidative stress, several cell types (including astrocytes) can activate the nuclear factor erythroid 2-related factor 2 (Nrf2), a regulator of several phase II detoxifying and antioxidant genes, such as the System X_c_^−^ subunit xCT. Here, we studied (i) the effect of the Aβ fragment 25-35 (Aβ_25-35_) on Nrf2-dependent System X_c_^−^ expression in U373 human astroglial cells and (ii) the effect of Aβ_25-35_-induced astrocytic response on neuronal cell viability using an in vitro co-culture system. We found that Aβ_25-35_ was able to activate an antioxidant response in astrocytes, by inducing both Nrf2 activation and System X_c_^−^ up-regulation. However, this astrocytic response caused an enhanced cell mortality of co-cultured SH-SY5Y cells, taken as a neuronal model. Consistently, the specific System X_c_^−^ inhibitor sulfasalazine prevented the increase of both neuronal mortality and extracellular glutamate levels, thus indicating that the neurotoxic effect was due to an augmented release of glutamate through the transporter. The involvement of NMDA receptor activation in this pathway was also demonstrated using the specific inhibitor MK801 that completely restored neuronal viability at the control levels. The present study sheds light on the Nrf2/system X_c_^−^ pathway in the toxicity induced by Aβ_25-35_ and may help to better understand the involvement of astrocytes in neuronal death during Alzheimer’s disease.

## 1. Introduction

Alzheimer’s disease (AD) is recognized by World Health Organization as a global public health priority, being the leading cause of dementia, responsible for 50–75% of all world cases and results in the deterioration of selective cognitive performance, including memory and mental processing [1,2].

AD is characterized by deposition of amyloid-β peptide (Aβ) in senile plaques, intracellular neurofibrillary tangles consisting of hyper-phosphorylated tau, synaptic dysfunction and neuronal death. Some or all of these hallmarks are causally linked to the cognitive and behavioral deficits that denote this disease [1,3,4].

Although the molecular mechanisms leading to neuronal damage in AD have not been completely understood, it is well established that accumulation of Aβ, in soluble and/or aggregated form, is a key pathogenetic event for AD [5,6]. The predominant forms of Aβ in the human brain as well as in human cerebrospinal fluid (CSF) are the full length Aβ_1-40_ and Aβ_1-42_ peptides and shorter carboxyterminal Aβ peptides, as well as amino-terminal truncated species [7]. In senile plaques, the predominant N-terminal truncated Aβ peptides are Aβ_3-40/42_, Aβ_11-40/42_, and Aβ_17-40/42_ [7]. Note that many Aβ fragments (e.g., Aβ_1-16_, Aβ_1-33_, Aβ_1-39_) in CSF can discriminate AD patients from non-demented controls [8]. Interestingly, Aβ fragment 25-35 (Aβ_25-35_) is the shortest fragment that exhibits large β-sheet fibrils and retains the toxicity of the full-length peptide [9]. Aβ_25-35_, physiologically present in elderly people, is the more toxic region and has been found to play a relevant role in free radical-associated neurotoxicity in AD, due to its peculiar aggregation properties [9].

Most of the known genetic, medical, environmental, and lifestyle-related risk factors for AD are associated with increased oxidative stress [10,11]. Indeed, AD brain is under strong oxidative stress, manifested by increased protein and DNA oxidation, lipid peroxidation, free radical formation, nitro-tyrosine levels, and advanced glycation end products [12,13,14]. Noteworthy, we previously demonstrated that both Aβ_25-35_ and Aβ_1-42_ are able to induce oxidative stress in endothelial cells, by producing superoxide and hydroxyl radicals [15]. Moreover, Aβ can form pore in astrocytes membranes and allow the influx of calcium from the extracellular space [16]. This modulation of calcium levels can induce the activation of NADPH oxidase and the subsequent reactive oxygen species (ROS) production, thus leading to Aβ-induced oxidative stress in astrocytes [17,18]. Chronic oxidative stress conditions arise because of an imbalance between the production of pro-oxidant molecules (e.g., ROS) and antioxidant system (e.g., intracellular glutathione (GSH) production), in favor of pro-oxidant molecules [19].

During oxidative stress, a variety of cell types are able to up-regulate the activity of nuclear factor erythroid 2-related factor 2 (Nrf2), the main regulator of the antioxidant response, thus counteracting intracellular ROS accumulation and GSH depletion [20,21]. Unlike neurons, astrocytes can strongly up-regulate Nrf2-mediated gene expression, thus leading to a major resistance to oxidative injury than isolated neurons [22]. Upon changes in cellular redox state, Nrf2 migrates to the nucleus and successively binds to promoter regions, known as antioxidant responsive element (ARE), of many phase II detoxifying and antioxidant genes, such as catalase (CAT), γ-glutamyl-cysteine ligase (GCL), superoxide dismutase (SOD), heme-oxygenase-1 (HO-1), glutathione peroxidase (GPX), and System X_c_^−^ subunit xCT [23,24]. System X_c_^−^ is an amino acid antiporter and mediates the exchange of intracellular L-glutamate and extracellular L-cystine across the plasma membrane [25]. In astrocytes, L-cystine import through System X_c_^−^ is crucial to glutathione production and protection from oxidative stress.

On the other side, however, glutamate export is a further route of release through which this neurotransmitter may provoke excitotoxicity [26,27]. Thus, System X_c_^−^ has currently been related to both pathological and physiological processes in the central nervous system (CNS) [28]. Even though the induction of Nrf2-dependent gene expression has been commonly reported as a protective mechanism to withstand the effects of oxidative stress in astrocytes, the up-regulation of xCT induced by Nrf2 could be a possible source for excitotoxicity due to excessive release of glutamate [29].

Despite many clinical and experimental data suggesting astrocytes as the cell population liable for most of the neuronal death in several neurodegenerative disorders, the specific cellular mechanisms are not yet clearly defined.

Here, we studied the effect of the Aβ_25-35_ on the induction of astroglial antioxidant response, focusing on the activation of Nrf2 transcription factor and on System X_c_^−^ expression. We also analyzed the effect of astrocytic System X_c_^−^ upregulation on the viability of neuronal cells in a co-culture system.

## 2. Materials and Methods

### 2.1. Materials

Amyloid-β Protein fragment 25-35 (Aβ_25-35_), DMEM (Dulbecco’s modified Eagle’s medium), FBS (fetal bovine serum), Trypsin–EDTA 0.25% solution, gentamicin 50 mg/mL solution, sulfasalazine (SSZ; a specific inhibitor of System X_c_^−^), MK-801 hydrogen maleate (MK-801; an NMDA receptor antagonist), and a kit for MTT assay were obtained from Sigma–Aldrich (Milan, Italy). The reagent for Bradford assay was from Bio-Rad Italia (Milan, Italy). All chemicals were of reagent or analytical grade and were used without further purification. TRIzol Reagent was from Life technologies Italia-Invitrogen, (Monza, Italy). The kit Go Taq 2-Step RT-qPCR System was obtained from Promega (Promega Italia Srl, Milan, Italy). For Western blot analysis and immunofluorescence, the following primary antibodies were used: anti-actin 1:1000 (a2066 Sigma-Aldrich; Milan, Italy), anti-Nrf2 (ab31163 Abcam), anti-Lamin A (ab26300 Abcam; Milan, Italy), anti-System X_c_^−^ (TA301518 OriGene; Bologna, Italy). For Western blot, secondary peroxidase-labeled anti-rabbit IgG antibodies were from Bio-Rad Italia (Milan, Italy). For immunofluorescence, secondary anti-Rabbit IgG Alexa Fluor 488 was obtained from Invitrogen. Hoechst 33342 (Cod. H3570) was from Thermofisher Scientific.

### 2.2. Cell Cultures and Treatments

U373-MG human glioblastoma astrocytoma cells and SH-SY5Y human neuroblastoma cells were purchased from ATCC (Manassas, VA, USA). Cells were grown in DMEM supplemented with 2 mM L-glutamine, 10% FBS and 40 μg/mL gentamicin at 37 °C in a humidified 5% CO_2_ incubator. Confluent monolayers of U373 cells were sub-cultured by conventional trypsinization. For the experiments, 2.5 × 10^5^ or 4 × 10^5^ cells were seeded in 35 or 60 mm tissue culture dishes, respectively, and grown up to 80% confluence for 18–24 h before treatments. Stock solution of Aβ_25-35_ was prepared at 2.5 mM concentration in bi-distilled water and kept frozen at −20 °C. For aggregation, Aβ_25–35_ was aged overnight at 25 °C before being added to the culture medium to the final desired concentration (for further details on Aβ_25–35_ preparation, conformation and aggregation properties see [9,30]).

### 2.3. SH-SY5Y Cell Differentiation

SH-SY5Y cells were seeded in a confluent monolayer in culture dishes established for the experiments. For neuronal differentiation, cells were cultured for a week in Neurobasal medium (Gibco; Milan, Italy) supplemented with 2 mM L-glutamine, 10 µM Retinoic Acid (Sigma, Milan, Italy) and 1X B-27 supplement (Gibco). The medium was changed every two days.

### 2.4. MTT Assay

To test neuronal viability, differentiated SH-SY5Y cells were grown alone and in co-cultures with U373 using a Transwell culture system as previously reported [29]. For each sample in co-cultures, 1.5 × 10^4^ neuronal cells were seeded in Transwell insert and 3 × 10^4^ astroglial cells were plated in the lower compartment of a 6-well plate and allowed to grow for 24 h.

MTT assay was performed at the end of the incubation period as indicated by manufacturer’s instructions and as reported elsewhere [29].

### 2.5. Quantitative Real-Time Reverse Transcription–Polymerase Chain Reaction

Total RNA was purified by using TRIzol Reagent and reverse transcribed with GoTaq 2-step RT-qPCR system. cDNA was then amplified for the following genes: System X_c_^−^ (xCT subunit; NM_014331.4), superoxide dismutase (SOD1; NM_000454.5 and SOD2; NM_000636.4), heme-oxygenase-1 (HO-1; NM_002133.3), catalase (CAT; NM_001752), glutathione peroxidase-3 (GPX3; NM_001329790.2), and glutamate-cysteine ligase (GCLC; AB262176.1). mRNA for Glyceraldehyde 3-phosphate dehydrogenase (GAPDH; NM_002046.7) was examined as the reference cellular transcript. The sequences of primers used for RT-qPCR were reported elsewhere [29]. The SYBR-Green method was applied to calculate PCR product quantification. Reactions were performed in an Agilent Aria Mx machine (Agilent technologies) using the following program: 45 cycles of 95 °C for 15 s, 60° C for 60 s, 72 °C for 20 s. GAPDH mRNA amplification products were present at equivalent levels in all cell lysates. Values were calculated relative to the internal housekeeping gene according to the second derivative test (delta–delta Ct (2^−ΔΔCT^) method).

### 2.6. Preparation of Nuclear and Total Extracts

After treatments, the cells were mechanically detached with a scraper in cold PBS. Nuclear extracts were prepared as reported elsewhere [29]. The protein content of nuclear extracts was measured according to Bradford method [31]. The quality of fraction separation was verified by blotting nuclear and cytosolic fractions for the specific markers, lamin A and actin, respectively. Total extracts were prepared by mechanically detaching the cells with a scraper in cold PBS. Extracts were then prepared as reported in [29]. The total protein content was determined according to Bradford method [31].

### 2.7. Evaluation of Nrf2 Activation and System X_c_^−^ Expression by Western Blotting

To measure Nrf2 nuclear levels, equal amounts of nuclear extracts (20 μg proteins/sample) were loaded in an 8% polyacrylamide gel, subjected to electrophoresis and transferred to nitrocellulose. After incubation with 5% non-fat dry milk for 1 h, membranes were incubated at 4 °C overnight with the polyclonal anti-Nrf2 antibody (1:1000) or with a polyclonal anti-lamin A (1:1000). To analyze System X_c_^−^ expression, equal amounts of total extracts (15 μg proteins/sample) were subjected to SDS-PAGE. Electrophoresis was performed using a 10% polyacrylamide gel. Membranes were blotted with anti-xCT polyclonal antibody (1:5000) or polyclonal anti-actin antibody (1:1000).

Actin and lamin A were used as reference proteins for total and nuclear extracts, respectively. Anti-rabbit secondary antibody labeled with peroxidase was used at 1:10,000 dilution. ECL Western blotting detection reagents was used to detect immunoreactive bands that were captured by Chemi Doc TM XRS 2015 (Bio-Rad Laboratories, Hercules, CA, USA). Densitometric analysis was carried out using Image Lab software (Version 5.2.1; © Bio-Rad Laboratories).

### 2.8. Analysis of Glutamate Concentration in Cell Supernatants

The release of glutamate in co-culture supernatants was evaluated using Glutamate Assay kit (BioVision; Florence, Italy), as reported elsewhere [29]. The concentration of glutamate in each sample was estimated using glutamate standard curve.

### 2.9. Immunofluorescence Analysis

1.5 × 10^5^ U373 cells were seeded on 6-well dishes containing poly-L-lysine-treated glass coverslip. After treatments, cells were washed in PBS and fixed with 4% paraformaldehyde for 10 min at room temperature (RT) and permeabilized in methanol for 10 min at −20 °C. Cells were then washed and incubated for 1 h at RT with a blocking solution (5% FBS, 1% BSA in PBS). Next, coverslips were incubated overnight at 4 °C with polyclonal anti-System X_c_^−^ (1:100). The secondary antibody anti-Rabbit IgG conjugated with Alexa Fluor 488 was diluted 1:500 and incubated at RT for 1 h. The nuclei were counterstained using Hoechst 33342 (Cod. H3570, Invitrogen; Milan, Italy, Thermofisher Scientific).

### 2.10. Statistical Analysis

Values are expressed as the mean ± standard error of the mean (SEM) of n observations. Statistical analysis was carried out by one-way ANOVA and subsequently by Bonferroni post-test. Differences are considered statistically significant at *p* ≤ 0.05.

## 3. Results and Discussion

Free radical production and oxidative stress play crucial roles in many neurodegenerative diseases, including Alzheimer’s disease [12]. In many cell types, including astrocytes, ROS can activate a protective antioxidant response through Nrf2-mediated induction of antioxidant and phase II detoxifying genes (i.e., ARE genes).

### 3.1. Aβ_25-35_ Activates Nrf2 in Astroglial Cells

**Figure 1:**
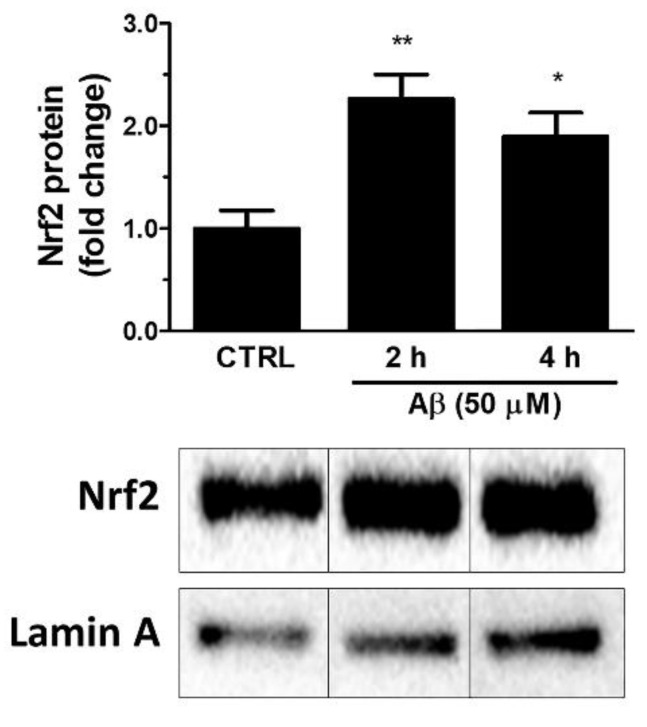
Effects of Aβ_25-35_ on nuclear translocation of Nrf2 in U373 cells. Cells were treated with Aβ_25-35_ (50 µM) for 2 and 4 h. The histograms show the densitometric analysis of the Western blots for each sample. Data are calculated relative to the housekeeping gene (i.e., nuclear lamin A) content and are the means ± SEM from three separate experiments, each performed in duplicate. One-way ANOVA, followed by Bonferroni’s test, was used to define significant differences. * *p* ≤ 0.05 vs. CTRL; ** *p* ≤ 0.01 vs. CTRL.

Firstly, we investigated whether Aβ_25-35_ could activate Nrf2 in astroglial cells. To this aim, U373 cells were treated with Aβ_25-35_ (50 μM) for 2, 4 and 24 h and Nrf2 levels were measured in nuclear extracts by Western blot analysis. The results shown in Figure 1 indicate that Aβ_25-35_ induced a 2.3-fold increase of the nuclear Nrf2 levels already at 2 h post-treatment and a 1.9-fold increase after 4 h of treatment.

### 3.2. Aβ_25-35_ Induces ARE Gene Expression in Astroglial Cells

**Figure 2:**
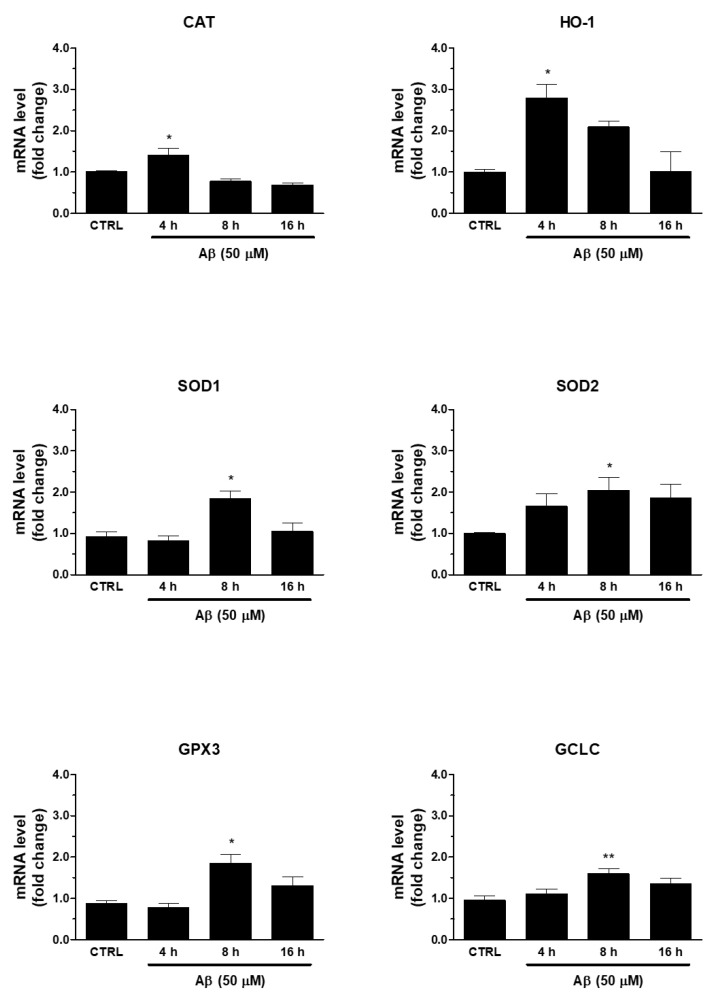
Effects of Aβ_25-35_ on ARE-dependent gene expression in U373 cells. Cells were treated with Aβ_25-35_ (50 µM) for 4, 8, and 16 h. The samples were then analyzed by RT-qPCR to evaluate the mRNA expression of CAT, HO-1, SOD1, SOD2, GCLC and GPX3 genes. Data are calculated relative to GAPDH content, taken as an internal housekeeping gene. Bars are the means ± SEM from three separate experiments, each carried out in duplicate. One-way ANOVA, followed by Bonferroni’s test, was used to define significant differences. * *p* ≤ 0.05 vs. CTRL; ** *p* ≤ 0.01 vs. CTRL.

Secondly, we verified whether Aβ-induced Nrf2 was able to regulate antioxidant ARE genes. In this respect, we found that the treatment of human U373 astroglial cells with Aβ_25-35_ (50 μM) for 4, 8, and 16 h was able to increase the mRNA expression of enzymes involved in maintenance of redox state, such as SOD1, SOD2, CAT, HO-1, GPX3, and GCLC (Figure 2). The peak was reached at 8 h for SOD1, SOD2, GPX3, and GCLC, whereas CAT and HO-1 expression peaked at 4 h after Aβ_25-35_ treatment. This time frame was compatible with the earlier transcriptional activation of Nrf2.

### 3.3. Aβ_25-35_ Induces System X_c_^−^ in Astroglial Cells

**Figure 3:**
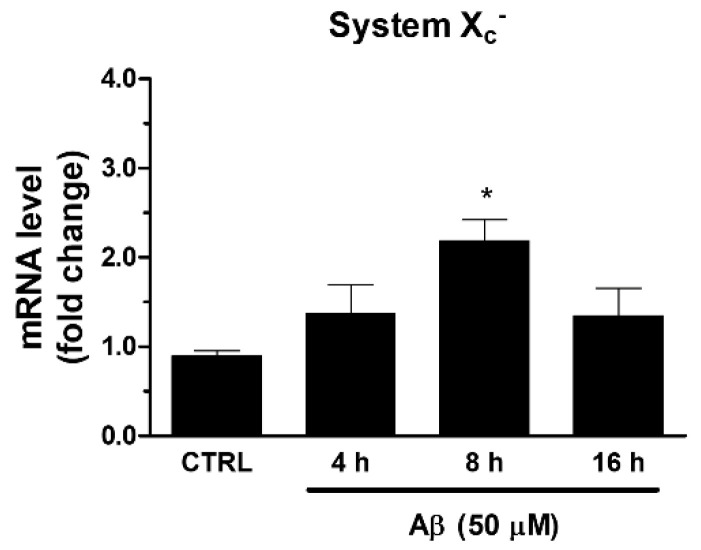
Effects of Aβ_25-35_ on System X_c_^−^ gene expression in U373 cells. Cells were treated with Aβ_25-35_ (50 µM) for 4, 8, and 16 h. After incubation at 37 °C, cells were homogenized, and total RNA has been purified to evaluate mRNA content of System X_c_^−^ by RT-qPCR. Results are computed relative to GAPDH content, taken as a housekeeping gene. Bars are the means ± SEM from three separate experiments, each performed in duplicate. One-way ANOVA, followed by Bonferroni’s test, was used to define significant differences. * *p* ≤ 0.05 vs. CTRL.

Among ARE genes, we further focused our attention on System X_c_^−^, which is involved in the maintenance of GSH intracellular levels and the redox state. In the same experimental conditions described above, we found that Aβ_25-35_ was able to increase the mRNA expression of xCT, the catalytic subunit of System X_c_^−^. As shown in Figure 3, we observed a maximum reached at 8 h post-treatment.

**Figure 4:**
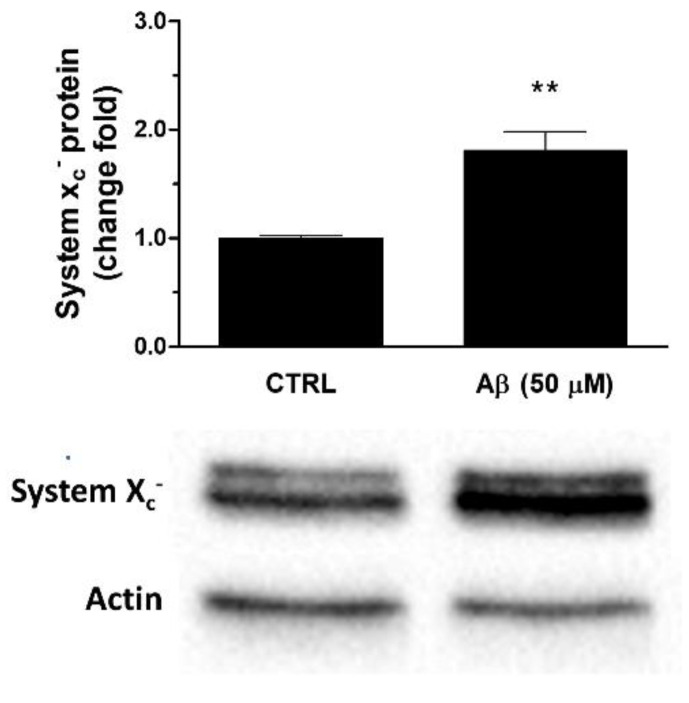
Effects of Aβ_25-35_ on System X_c_^−^ protein expression in U373 cells. Cells were treated with Aβ_25-35_ (50 µM) for 24 h. The graph shows the densitometric analysis of the western blots for each sample. Data are computed relative to the internal housekeeping gene (actin) and are the means ± SEM from three separate experiments, each carried out in duplicate. One-way ANOVA, followed by Bonferroni’s test, was used to define significant differences. ** *p* ≤ 0.01 vs. CTRL.

Consistently, we observed that Aβ_25-35_ was able to up-regulate System X_c_^−^ also at protein level. In particular, a 24 h treatment of U373 cells with Aβ_25-35_ (50 μM) caused a two-fold increase of System X_c_^−^ protein levels in whole cell extracts when compared to controls, as verified by Western blot analyses (see Figure 4).

**Figure 5:**
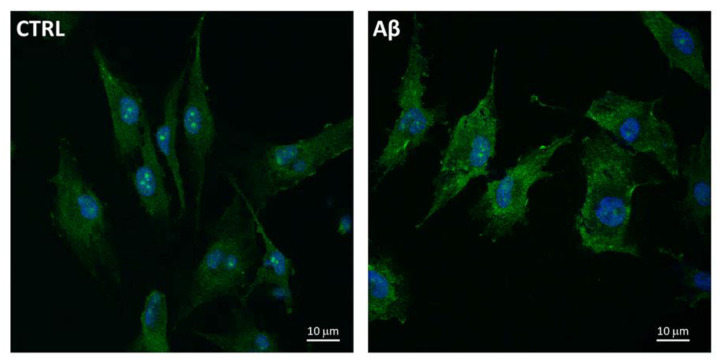
System X_c_^−^ localization in Aβ_25-35_-treated U373. Cells were treated with Aβ_25-35_ (50 µM) for 24 h and subjected to immunofluorescence staining using anti-System X_c_^−^ 1:100 (OriGene, green) antibodies as reported in Materials and Methods. Nuclei (blue) are stained with Hoechst 33342.

Furthermore, we used the confocal microscopy to also evaluate the expression and localization of System X_c_^−^ in U373 cells treated with Aβ_25-35_ (50 μM) for 24 h. Figure 5 shows an increased levels of System X_c_^−^, the latter being mainly localized on plasma membrane of treated cells compared to controls at 24 h post-treatment.

### 3.4. Aβ_25-35_ Induces Glutamate Release through System X_c_^−^

The above results would seem to be in agreement with the concept that astrocytes play an important role in providing antioxidant support to neighboring neurons. In fact, post-mitotic neurons are thought to survive for many decades despite their relatively low intrinsic antioxidant defenses [22,32,33,34]. Given the role of System X_c_^−^ in providing the cell with cystine, its augmented expression and/or activity increases intracellular levels of cysteine. That is the rate-limiting substrate for the synthesis of GSH, thereby endowing astrocytes with an effective antioxidant response.

**Figure 6:**
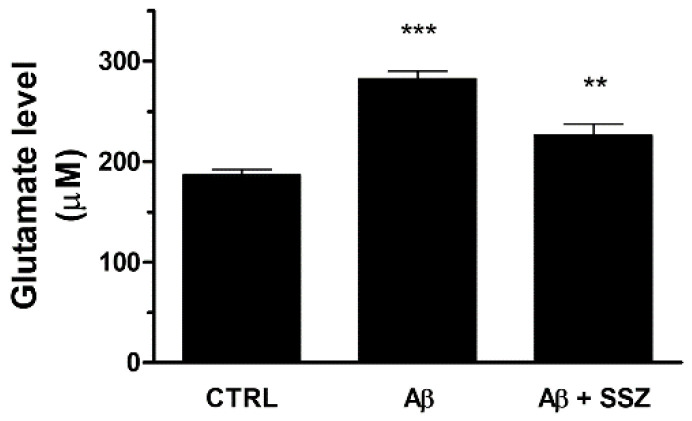
Effects of Aβ_25-35_ and System X_c_^−^ activity on release of extracellular glutamate in differentiated SH-SY5Y/U373 co-cultures. Cells were treated for 24 h and the glutamate assay was performed as specified in the Materials and Methods. The graph shows the extracellular release of glutamate (µM). Data are calculated relative to a glutamate standard curve and are the means ± SEM from three separate experiments, each carried out in duplicate. One-way ANOVA, followed by Bonferroni’s test, was used to define significant differences. *** *p* ≤ 0.001 between CTRL and Aβ_25-35_; ** *p* < 0.01 between Aβ_25-35_ and Aβ_25-35_ + SSZ.

Nevertheless, the up-regulation of System X_c_^−^ can increase extracellular glutamate release and potentially cause excitotoxicity. To verify whether the treatment of astrocytes with Aβ_25-35_ can enhance the release of glutamate in the extracellular space, we quantified the levels of glutamate in the supernatant of U373 co-cultured with differentiated SH-SY5Y cells in the presence of Aβ_25-35_ (50 μM) for 24 h. As shown in Figure 6, Aβ_25-35_-treated cells released about 50% more glutamate with respect to untreated cells. As a control, we found that glutamate was also released from mono-cultured U373 cells treated with Aβ_25-35_ for 24 h, thus proving its astroglial origin (data not shown).

To confirm that Aβ_25-35_-elicited glutamate release occurred through System X_c_^−^ activation, we analyzed the levels of glutamate in the supernatants of co-cultures in the presence of sulfasalazine (SSZ; 300 μM), a specific inhibitor of System X_c_^−^. We observed that SSZ treatment prevented Aβ_25-35_-induced glutamate release, reducing its levels in the extracellular space. These data clearly demonstrate that in astroglial cells the treatment with Aβ_25-35_ increases the release of glutamate by eliciting System X_c_^−^ up-regulation (see Figure 6).

### 3.5. Aβ_25-35_ Affects Viability of Co-Cultured SH-SY5Y Cells via System Xc^−^ and NMDA Receptor

**Figure 7:**
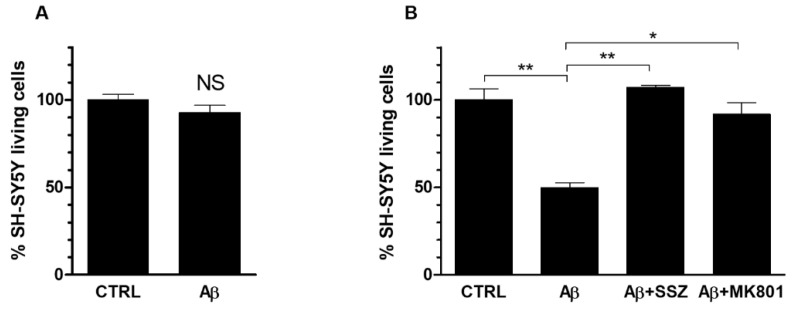
Role of System X_c_^−^ and NMDA receptor activation on the viability of Aβ-treated SH-SY5Y differentiated cells. (**A**) SH-SY5Y cells were grown in mono-cultures and treated for 24 h with Aβ_25-35_ (50 μM). (**B**) SH-SY5Y cells were co-cultured with U373 cells and treated for 24 h with Aβ_25-35_ (50 μM) alone or in the presence of either SSZ (300 μM) or MK801 (10 μM). MTT cell viability assay was carried out as specified in Materials and Methods. The histograms show the percentage of living cells, and the rate of reduction was calculated by setting the control (CTRL) equal to 100%. Values are the means ± SEM from three separate experiments, each performed in duplicate. One-way ANOVA, followed by Bonferroni’s test, was used to determine significant differences. NS not significant; ** *p* ≤ 0.01 between CTRL and Aβ_25-35_; ** *p* ≤ 0.01 between Aβ_25-35_ and Aβ_25-35_ + SSZ; * *p* ≤ 0.05 between Aβ_25-35_ and Aβ_25-35_ + MK801.

To verify whether the System X_c_^−^-mediated increase of glutamate release by astroglial cells caused neurotoxicity, we investigated the viability of neurons cocultured with astrocytes in the presence of Aβ_25-35_. First of all, we verified that a 24 h treatment with Aβ_25-35_ (50 μM) of differentiated SH-SY5Y cells, cultured alone, did not affected cell viability (Figure 7a). Afterwards, we evaluated the viability of neuronal cells grown in co-cultures with astrocytes, in the presence of Aβ_25-35_. As shown in Figure 7b, a treatment with Aβ_25-35_ for 24 h caused a significant reduction of 50% less viability of differentiated neuronal SH-SY5Y cells co-cultured with U373 cells in comparison to untreated cells. To verify whether Aβ_25-35_-induced neurotoxicity was due to an enhancement of glutamate export through System X_c_^−^, we carried out the MTT assay in co-cultures treated with Aβ_25-35_ for 24 h in the presence of SSZ (300 μM). Our results indicate that SSZ prevented neurotoxicity in SH-SY5Y cells co-cultured with U373 cells, thus restoring neuronal viability at the control level (see Figure 7b). These data suggest that Aβ_25-35_-induced neurotoxic effect is mediated by increased glutamate release due to System X_c_^−^ up-regulation in astroglial cells.

The release of glutamate via System X_c_^−^ from both microglia and astrocytes has been found to increase excitotoxicity of cortical neurons [35,36,37,38,39,40]. Noteworthy, changes in glutamate transport have been demonstrated in a mouse model for Alzheimer’s disease. Enhanced cortical expression of VGLUT3 and xCT along with a strong trend towards increased cortical extracellular glutamate levels have been reported elsewhere [41]. In neurons, excitotoxicity occurs via glutamate-induced overactivation of NMDA receptor and subsequent perturbed cellular calcium homeostasis and mitochondrial alterations [42,43].

To assess whether the reduced neuronal viability, as triggered by extracellular glutamate release through System X_c_^−^, was effectively due to the activation of NMDA receptor, we performed MTT assay on SH-SY5Y (grown in co-cultures with U373 cells) treated for 24 h with Aβ_25-35_ in the presence of MK801 (10 μM), an NMDA receptor antagonist. As shown in Figure 7b, MK801 prevented neuronal toxicity restoring the percentage of neuronal living cells at the control level. These results clearly indicate that Aβ_25-35_-induced neurotoxicity is mediated by the activation of NMDA receptor elicited by System X_c_^−^-dependent glutamate release.

Note that during neuroinflammation, activated astrocytes and microglia have been reported to release and maintain high concentrations of extracellular glutamate [40]. Moreover, excitotoxic glutamate release and high levels of System X_c_^−^ expression were observed in microglia, due to a prolonged need for oxidative protection [44]. Interestingly, neurons co-cultured with astrocytes were observed to be more susceptible than neurons alone to hypoxic cell death after treatment with IL-1β, an effect being mediated by enhanced glutamate efflux from astrocytes through System X_c_^−^ [36]. Very recently, we have reported that HIV-1 Tat protein was able to induce neurotoxicity by eliciting Nrf2-mediated System X_c_^−^ activation [29]. Previously, we reported that HIV-1 Tat can induce neuro-toxicity by eliciting the spermine oxidase-dependent ROS generation through NMDA receptor stimulation in SH-SY5Y cells, which in turn leads to GSH depletion and oxidative stress [45]. Finally, we have recently demonstrated that System X_c_^−^ participated in the increase of glutamate excitotoxicity in the neocortex of a mouse model (Dach-SMOX), displaying a chronic oxidative stress [46].

Although Aβ_25-35_ may have some limits in representing the whole Aβ peptide and is a scarce version in vivo, it is particularly worthy of attention in light of its great oxidative stress generation capacity and extreme toxicity in neuronal cells and synaptosomes [9]. Altogether, our data show how inflammatory pathways and oxidative stress may converge to an intersection point, represented by activation of System X_c_^−^, thereby suggesting a possible explanation for the mechanism involved in excitotoxicity induced by Aβ_25-35_. It should be pointed out, however, that Aβ_25-35_ is just one of the fragments and this does not exclude the possibility that other parts of Aβ, than the 25-35 fragment, can be involved in the induction of Nrf2/SystemX_c_^−^ pathway.

## 4. Conclusions

**Figure 8:**
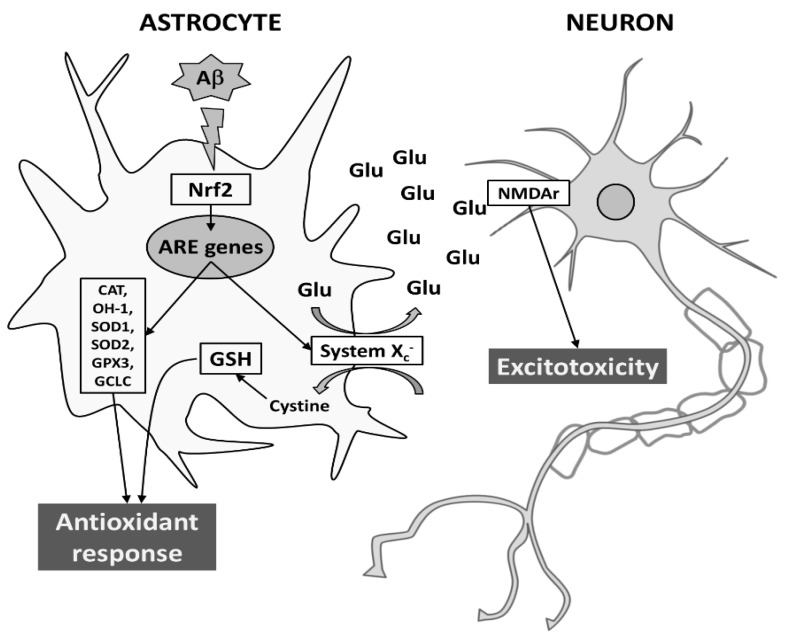
Proposed model for Aβ-dependent neurodegeneration during AD. In astrocytes, Aβ_25-35_ elicits an antioxidant response by transcriptionally inducing Nrf2-driven ARE genes, such as CAT, HO-1, SOD1, SOD2, GPX3, GCLC, and System X_c_^−^. While the L-cystine import through System X_c_^−^ is crucial to protection from oxidative stress (e.g., GSH production), the export of glutamate may cause neurodegeneration through the activation of NMDAr on neuronal cells. For more details see text.Abbreviations: Aβ, amyloid-β; ARE, antioxidant responsive element; CAT, catalase; GCLC, glutamate-cysteine ligase; GPX3, glutathione peroxidase; GSH, reduced glutathione; HO-1, heme-oxygenase-1; NMDAr, N-methyl-D-aspartate receptor; Nrf2, nuclear factor erythroid 2-related factor 2; SOD, superoxide dismutase.

Given the role played by astrocytes in maintaining the homeostasis of extracellular space in the brain, their response may affect neuronal function and provide antioxidant support to neighboring neurons both in normal and pathological conditions. Here, we show that Aβ_25-35_ is able to trigger an antioxidant response in astrocytes, by inducing both Nrf2 and ARE-driven genes, including System X_c_^−^. However, the induction of System X_c_^−^ in astrocytes seems to be also responsible for mortality of neuronal cells, due to sustained glutamate release and neuronal NMDA receptor activation. Although further studies are needed, it is tempting to speculate that neurodegeneration can be exacerbated by converting oxidative stress to excitotoxicity via System X_c_^−^ (for a schematic model see Figure 8).

In conclusion, the present study highlights the importance of the Nrf2/System X_c_^−^ pathway for a better understanding of the role of astrocytes as a cell population responsible for the death of neurons in AD.

## Data Availability

Data is contained within the article.
